# Effect of high-quality nursing intervention on the psychological disorder in patients with gastric cancer during perioperative period: A protocol of systematic review and meta-analysis

**DOI:** 10.1097/MD.0000000000020381

**Published:** 2020-06-05

**Authors:** Xiu-Li He, Zhi-Min Cao

**Affiliations:** a Department of Nursing Care, Yan’an People's Hospital; b Department of Nursing Care, Yan’an Second People's Hospital, Yan’an, China.

**Keywords:** effect, gastric cancer, high-quality nursing intervention, psychological disorder, randomized controlled trial

## Abstract

**Background:**

This study will be proposed for investigating the effects of high-quality nursing intervention (HQNI) on the psychological disorder in patients with gastric cancer during perioperative period (GC-PPP).

**Methods:**

A cumulative search from inception up to the March 31, 2020 will be performed in the following databases: Cochrane Library, MEDLINE, EMBASE, Web of Science, VIP database, and China National Knowledge Infrastructure. We will search all potential studies from those electronic databases regardless their language and publication status. We will only consider randomized controlled trials (RCTs) for inclusion, which explores the effect of HQNI on the psychological disorder in patients with GC-PPP. Study identification, information extraction, and study quality appraisal will be independently and respectively done by 2 researchers. Any different opinions between 2 researchers will be disentangled by a third researcher after discussion. Cochrane risk of bias tool will be used for study quality assessment, and RevMan 5.3 software will be utilized for statistical analysis.

**Results:**

This study will provide a high-quality synthesis of psychological disorder outcomes to evaluate the effects and safety of HQNI for patients with GC-PPP.

**Conclusion:**

The findings of this study will provide reference and evidence to appraise whether HQNI is an effective on the psychological disorder in patients with GC-PPP

**Study registration number:**

INPLASY202040080.

## 1 Introduction

Gastric cancer (GC) remains one of the most common cancers, which is the leading cause of cancer-related death globally.^[1–4]^ Previous epidemiological study reported more than 1,000,000 new cases and 783,000 deaths of GC around the world.^[5,6]^ If it cannot be identified and treated at early stage, most patients result in very severe outcome results.^[7–9]^ Thus, it is very important to diagnose and to treat this condition at early stage.

Surgery is the most widely treatment for GC.^[10–12]^ However, most patients with gastric cancer during perioperative period (GC-PPP) experience psychological disorder.^[13–18]^ Fortunately, high-quality nursing intervention (HQNI) is reported to mange this condition effectively.^[19–24]^ It is still unclear whether HQNI on psychological disorder in patients with GC-PPP is effective and safe. Therefore, it is very necessary to systematically assess the effect of HQNI on psychological disorder in patients with GC-PPP, and to determine whether it is a good choice for such patients.

## 2 Methods

### 2.1 Study registration

We have registered this study through INPLASY202040080, and we have reported it following the guideline of the Preferred Reporting Items for Systematic Review and Meta-Analysis Protocols Statement.^[25]^

### 2.2 Ethics and dissemination

All data utilized in this study will be collected from previous trials. Thus, no ethic approval is needed. This study will be published through a peer-reviewed journal.

### 2.3 Criteria for included studies

#### 2.3.1 Study types

This proposed study will include randomized controlled trials (RCTs) reported for assessing the effects of HQNI on the psychological disorder in patients with GC-PPP with no restrictions of language and publication status. We will exclude any uncontrolled trials, non-RCTs and quasi-RCTs.

#### 2.3.2 Participants

All GC-PPP patients (18 years old or more) with psychological disorder, including depression and anxiety will be fully considered, regardless the race, gender, and country.

#### 2.3.3 Interventions

Experimental group: We will include all patients who received HQNI for the management of psychological disorder.

Control group: We will consider participants who underwent any treatments. However, we will exclude patients who also received any forms of HQNI.

#### 2.3.4 Outcomes

Primary outcomes are depression (as measured by any validated scales, such as Hamilton Depression Rating Scale), and anxiety (as assessed by any validated scores, such as Hamilton Anxiety Rating Scale).

Secondary outcomes consist of health-related quality of life (as checked by any relevant tools, such as 36-Item Short Form Health Survey), and incidence of any adverse events.

### 2.4 Strategy of literature searches

The following electronic databases will be retrieved cumulatively from inception up to the March 31, 2020: Cochrane Library, MEDLINE, EMBASE, Web of Science, VIP database, and China National Knowledge Infrastructure. The RCTs of HQNI for the management of psychological disorder in patients with GC-PPP will be searched from above databases. The literatures included will not subject to any language and publication status. The Cochrane Library search strategy is presented in Table 1.

**Table 1:**
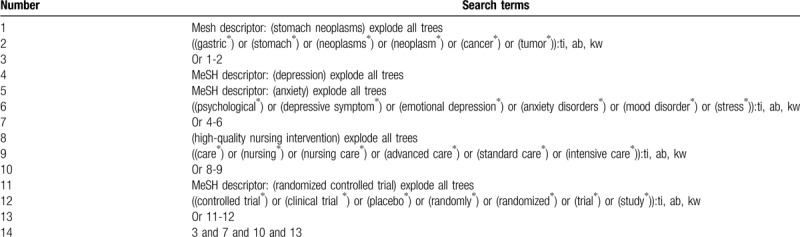
Search strategy of Cochrane Library.

In addition, a reference list of included RCTs and related reviews will be examined. We will search relevant conference abstracts and new trials from the clinical trial registry.

### 2.5 Data collection

#### 2.5.1 Study selection

Two researchers will import all searched records into Endnote X7 and all duplicate citations will be eliminated. The titles and abstracts of all searched literatures will be identified according to the eligibility criteria. All irrelevant studies will be excluded. The potential trials will be furthered assessed by reading the full-text papers. All excluded studies will be noted and listed in the table with specific reasons. Any differences will be solved by a third researcher through consultation. The selection of study process is presented in a flow diagram.

#### 2.5.2 Data extraction

All essential data will be collected by 2 independent researchers using a predefined data extraction sheet. Any inconsistent views will be figured out through discussion by a third researcher. This data extraction sheet will consist of study information, time of publication, first author, participants, diagnostic criteria, inclusion and exclusion criteria, randomization details, blind, allocation, interventions, comparators, indicators, findings, and adverse events. If any unclear or missing data is identified, we will contact the trial authors to obtain such information.

### 2.6 Assessment of risk of bias for included trials

Two independent researchers will assess the risk of bias for each included trial using

Cochrane Collaboration Tool through 7 items. Each item is graded as low, unclear, and high risk of bias. If any different opinions occur between 2 researchers, we will invite a third researcher to solve them by discussion.

### 2.7 Statistical analysis

We will apply ReMan 5.3 software to perform statistical analysis. We will express continuous values using mean difference or standardized mean difference and 95% confidence intervals, and dichotomous values using risk ratio and 95% confidence intervals. *I*^*2*^ test will be utilized to assess inconsistencies and heterogeneity across included trials. *I*^*2*^ ≤ 50% means a minor heterogeneity, and a fixed-effect model will be used to synthesize the outcome indicator data. *I*^*2*^ > 50% exerts substantial heterogeneity, and a random-effect model will be utilized to pool the outcome indicator data. If ample data are included for specific types of intervention and control, a meta-analysis will be undertaken if trials are sufficiently similar with respect to the study information, patient characteristics, interventions, controls, and outcome indicators. Otherwise, we will carry out subgroup analysis to identify possible sources of the significant heterogeneity.

#### 2.7.1 Subgroup analysis

Subgroup analysis will be conducted based on the different interventions, comparators, and outcome indicators to explore any possible sources of significant heterogeneity among included trials.

#### 2.7.2 Sensitivity analysis

If necessary, sensitivity analysis will be undertaken to investigate the robustness and stability of study findings by removing studies with high risk of bias.

#### 2.7.3 Reporting bias

We will also perform funnel plot^[26]^ and Egger regression test^[27]^ to check any possible reporting bias if at least 10 included trials are included.

#### 2.7.4 Grading the quality of evidence

We will evaluate the quality of evidence for each outcome indicator through the Grading of Recommendations Assessment Development and Evaluation approach.^[28]^ It rates as 5 levels of very low, low, medium, and high. Its results will be summarized in the "Summary of Findings” table.

## 3 Discussion

The purpose of this study is to assess the effect of HQNI on psychological disorder in patients with GC-PPP. To our best knowledge, this is the first study to investigate the effect of HQNI on psychological disorder in patients with GC-PPP. The conclusions drawn from this study may be beneficial to both patient and clinicians, and health-related policy makers. However, there are some potential limitations in this study. First, there may be a risk of heterogeneity in study quality, and an insufficient number of high quality RCTs. Second, there may be a small sample size of included studies. Finally, there may be missing potential eligible studies, although we try our best to search more electronic databases and grey literatures.

## Author contributions

**Conceptualization:** Xiu-Li He, Zhi-Min Cao.

**Data curation:** Xiu-Li He, Zhi-Min Cao.

**Formal analysis:** Xiu-Li He, Zhi-Min Cao.

**Investigation:** Zhi-Min Cao.

**Methodology:** Xiu-Li He.

**Project administration:** Zhi-Min Cao.

**Resources:** Xiu-Li He.

**Software:** Xiu-Li He.

**Supervision:** Zhi-Min Cao.

**Validation:** Xiu-Li He, Zhi-Min Cao.

**Visualization:** Xiu-Li He, Zhi-Min Cao.

**Writing – original draft:** Xiu-Li He, Zhi-Min Cao.

**Writing – review & editing:** Xiu-Li He, Zhi-Min Cao.
